# Discovery and Optimization of the Novel Immunotoxins Targeting TROP2 for Cancer Therapy

**DOI:** 10.3390/ph19101586

**Published:** 2026-10-07

**Authors:** Junyan Li, Tanja Poredoš, Shuyi Xu, Lei Wang, Haiyang Yin, Yali Yue, Junjun Liu, Tong Wu, Yanlin Bian, Yueqing Xie, Jianwei Zhu

**Affiliations:** 1 Engineering Research Center of Cell & Therapeutic Antibody, School of Pharmacy, Shanghai Jiao Tong University, Ministry of Education, Shanghai 200240, China; sjtu.ljy@sjtu.edu.cn (J.L.); tanja.jakos@gmail.com (T.P.);; 2 Jecho Institute Co., Ltd., Shanghai 200240, China; 3 Jecho Laboratories, Inc., Frederick, MD 21704, USA

**Keywords:** immunotoxin, TROP2, *Pseudomonas* Exotoxin A, stability, dsFv, ABD

## Abstract

**Background:** Trophoblast cell surface antigen 2 (TROP2) is widely overexpressed in epithelial cancers and associated with poor prognosis, representing an attractive target for cancer treatment. Recent clinical studies reported favorable outcomes of TROP2-targeting antibody–drug conjugates. However, heterogeneity of human cancers and development of resistance encourage the exploration of alternative cytotoxic payloads. In this regard, bacterial immunotoxin is a viable treatment option owing to the unique and potent mechanism of action. **Methods**: We first constructed three immunotoxins by fusing deimmunized *Pseudomonas* Exotoxin A (PE24) to novel TROP2-targeting single-chain variable fragments identified through phage display, which were screened for high binding and cytotoxic activity against tumor cell lines with distinct TROP2 expression. The lead immunotoxin T3PE was engineered by introducing extra disulfide bonds and an albumin-binding domain (ABD) to generate disulfide-stabilized T3PE (dsT3PE and dsT3PE_ABD_CC). Then, both variants were characterized in stability, cell binding, internalization, pharmacokinetic profiles, and in vitro and in vivo antitumor activities. **Results**: T3PE bound strongly to TROP2 (EC_50_ = 0.3824 μg/mL) and exerted potent cytotoxicity against NCI-N87 cells (EC_50_ = 0.09074 μg/mL), but it displayed poor serum stability and was prone to aggregation. Introduction of disulfide bonds stabilized the immunotoxins while preserving the potent cytotoxic activity, and fusion of ABD extended the plasma half-life 6-fold in mice. Additionally, five doses of either dsT3PE or dsT3PE_ABD_CC at 3 mg/kg could significantly reduce tumor progression in the NCI-N87 mouse xenograft model. **Conclusions**: Together, these results demonstrated the therapeutic potential of these novel anti-TROP2 immunotoxins against TROP2-overexpressing tumors.

## 1. Introduction

Surgery, chemotherapy and radiotherapy have long been the primary treatment options for cancer. However, most cytotoxic drugs, as the mainstay of systemic treatment, lack tumor selectivity, leading to narrow therapeutic windows and toxicity to normal tissues [1,2]. In contrast, monoclonal antibodies (mAbs) offer high specificity, but their intrinsic cytotoxicity is often insufficient to eradicate tumors [1,2]. To address this issue, antibodies can be repurposed as delivery vehicles for cytotoxic payloads, thus improving their pharmacodynamic and pharmacokinetic (PK) properties [1,2]. This approach has been increasingly utilized in recent years, resulting in the approval of 21 antibody–drug conjugates (ADCs) and three recombinant immunotoxins (RITs). ADCs chemically link full IgG molecules to small-molecule cytotoxic drugs, and RITs genetically fuse antibody fragments or cytokine ligands to protein toxins [3,4]. *Pseudomonas* Exotoxin A (PE), one of the commonly used protein toxins, is a well-studied ADP-ribosyltransferase that prevents the synthesis of cellular proteins by ADP-ribosylating eukaryotic elongation factor 2 [3]. Compared with ADCs, RITs present several notable benefits. First, clinical-grade immunotoxins can be obtained through cost-effective prokaryotic expression platforms, as exemplified by the FDA-approved moxetumomab pasudotox produced in *E. coli* [5]. Moreover, the catalytic mechanism of action, which is independent from cell proliferation and allows the modification of many substrate molecules per toxin molecule, endows immunotoxins with remarkable potency and enables the killing of chemotherapy-resistant cells [6].

Despite these advantages and the fact that several RITs have received regulatory approval or entered clinical trials, critical challenges, including immunogenicity and non-specific toxicity, continue to hinder their broader clinical applications [2,7,8]. Both bacterial toxins and mouse-derived antibody sequences in early-generation immunotoxins represent major sources of immunogenicity and trigger anti-drug antibodies (ADAs). This is exemplified by moxetumomab pasudotox, an anti-CD22 immunotoxin, that comprises a murine single-chain variable fragment (scFv) fused to PE38 [9]. A phase III trial of moxetumomab pasudotox revealed the detection of ADAs directed against both the CD22-binding domain and the PE38 toxin moiety [3,10,11]. To address immunogenicity associated with antibody fragments, humanized or fully human antibody sequences have increasingly been employed in the construction of immunotoxins in recent years [12,13]. In addition, to reduce the immunogenicity, as well as the non-specific toxicity, truncated PE derivatives with various molecular sizes have been evaluated. Among these, PE24 represents one of the outstanding variants and retains only the catalytic domain and the furin cleavage site (FCS) [7,14]. Beyond domain truncation, seven amino acid exchanges were introduced into Domain III of PE to silence B-cell epitopes, substantially reducing immunogenicity while retaining antitumor activity [15]. Furthermore, the native C-terminal REDLK of PE was replaced with KDEL to facilitate endoplasmic reticulum transport and thereby increase cytotoxicity [16]. These engineering strategies have been incorporated into next-generation immunotoxins such as LMB-100, a mesothelin-targeted construct that has progressed to phase I/II clinical trials [17].

Alongside antibody and toxin engineering, target antigen selection represents an equally critical determinant of immunotoxin performance, directly influencing biodistribution, cellular uptake efficiency and on-target off-tumor toxicity. Trophoblast cell surface antigen 2 (TROP2) is a type I transmembrane glycoprotein with signal transducing activity, which has been observed to be overexpressed in a variety of epithelial cancers, including 62.4% of breast cancers (78.1% of triple-negative breast cancers (TNBCs)) and 66.3% of gastric cancers [18]. Evidence indicates that TROP2 can promote tumor cell proliferation, invasion and drug resistance and has been validated as an independent prognostic indicator of adverse outcomes [18,19,20]. Recent preclinical and clinical data confirmed TROP2 as a promising therapeutic target for cytotoxic payload delivery. TROP2-targeting immunotoxins, including 5G8-PE38 [21] and Fab-PE24 [22], have demonstrated potent antitumor activity in TROP2-positive tumor models, confirming that the efficient receptor-mediated internalization is sufficient to deliver enzymatic toxins to the cytosol. In addition, anti-TROP2 ADCs have shown remarkable clinical activity with manageable safety profiles, leading to the successive approvals of sacituzumab govitecan, sacituzumab tirumotecan and datopotamab deruxtecan [23,24,25,26]. However, all three anti-TROP2 ADCs carry topoisomerase I inhibitor payloads, raising concerns about payload-related resistance and cross-resistance upon sequential ADC administration [27,28]. In this context, the complementary cytotoxic mechanism of PE-based immunotoxins is particularly appealing. Moreover, PE-based immunotoxins are dose-limited primarily by capillary leak syndrome [17], whereas topoisomerase I inhibitor payloads are dominated by myelosuppression and interstitial lung disease [24,29,30]. This non-overlapping dose-limiting toxicity profile suggests a potential tolerability advantage upon sequential administration.

Taken together, the scientific evidence supports RITs as an innovative and promising therapeutic strategy for TROP2-overexpressing carcinomas. In this study, we identified a panel of TROP2-binding antibody sequences through phage display and constructed three scFv-PE24 immunotoxins, which were then screened for binding and cytotoxic activity. The lead candidate was selected for further optimization. Specifically, additional disulfide bonds were introduced to enhance structural stability and an albumin-binding domain (ABD) was fused to prolong plasma half-life in vivo. The optimized immunotoxins were comprehensively evaluated for stability, in vitro biological activity, PK profile and in vivo antitumor efficacy to assess the therapeutic potential.

## 2. Results

### 2.1. Design and Preparation of scFv-Based Recombinant Immunotoxins Targeting TROP2

**Figure 1:**
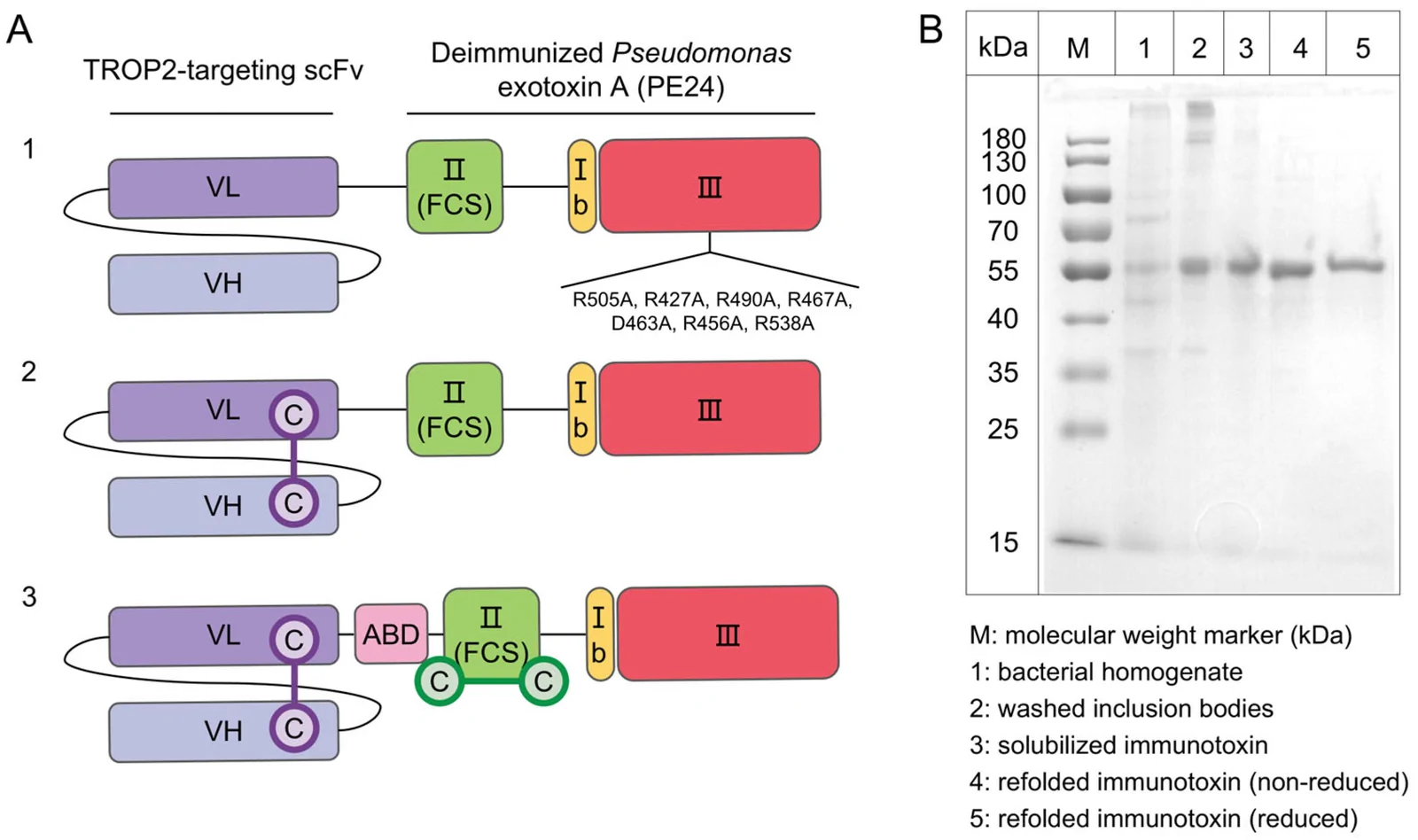
Design and generation of scFv-based anti-TROP2 immunotoxins. (**A**) Schematic diagram of the scFv-based and disulfide-stabilized immunotoxins. 1, scFv-based immunotoxin: the anti-TROP2 scFv (purple) was fused to the truncated and deimmunized variant of PE (PE24), which consisted of the flexible linker containing an FCS (green) and the catalytic fragment (the last 4 amino acids of domain Ib (orange) and the domain III (red): residue 400-613 in native PE). Seven surface-exposed residues that form immunogenic B-cell epitopes were mutated and the C-terminal REDLK motif was changed to KDEL. 2 and 3, disulfide-stabilized immunotoxins: C-C, a disulfide bond. ABD, albumin-binding domain (pink). (**B**) Representative SDS-PAGE analysis of immunotoxin expression and purification from *E. coli* inclusion bodies. Lane M, molecular weight marker (kDa); lane 1, crude bacterial homogenate; lane 2, enriched immunotoxin in washed inclusion bodies; lane 3, denatured immunotoxin after solubilization; lanes 4 (non-reduced) and 5 (reduced), refolded active immunotoxin after dialysis in PBS.

Human scFv sequences were isolated through phage display against recombinant human TROP2 protein. Thirty positive clones were converted into scFv-Fc format. The sequences showing weak binding signals to TROP2 protein or non-specific binding to the TROP2-negative cell line were excluded. Cross-competition epitope binning assay of the remaining eight candidates revealed three distinct epitope bins, and most candidates exhibited effective binding to TROP2 protein in an ELISA assay (Figure S1). One candidate sequence from each epitope bin (T3, T7 and T21) was selected for the construction of the scFv-based immunotoxin, named T3PE, T7PE and T21PE (Figure 1A). The immunotoxins were expressed in *E. coli* and enriched in the insoluble fraction. Following extensive inclusion body washing, solubilization and refolding, the immunotoxins were prepared with sufficient purity to permit preliminary evaluation of target binding, cell binding and cytotoxic activity (Figure 1B). The slight shift in protein band (lane 4) indicated successful structural rearrangement.

### 2.2. T3PE Showed Stronger Binding to Cell Lines with High TROP2 Expression and Demonstrated Favorable Cytotoxic Activity In Vitro

**Figure 2:**
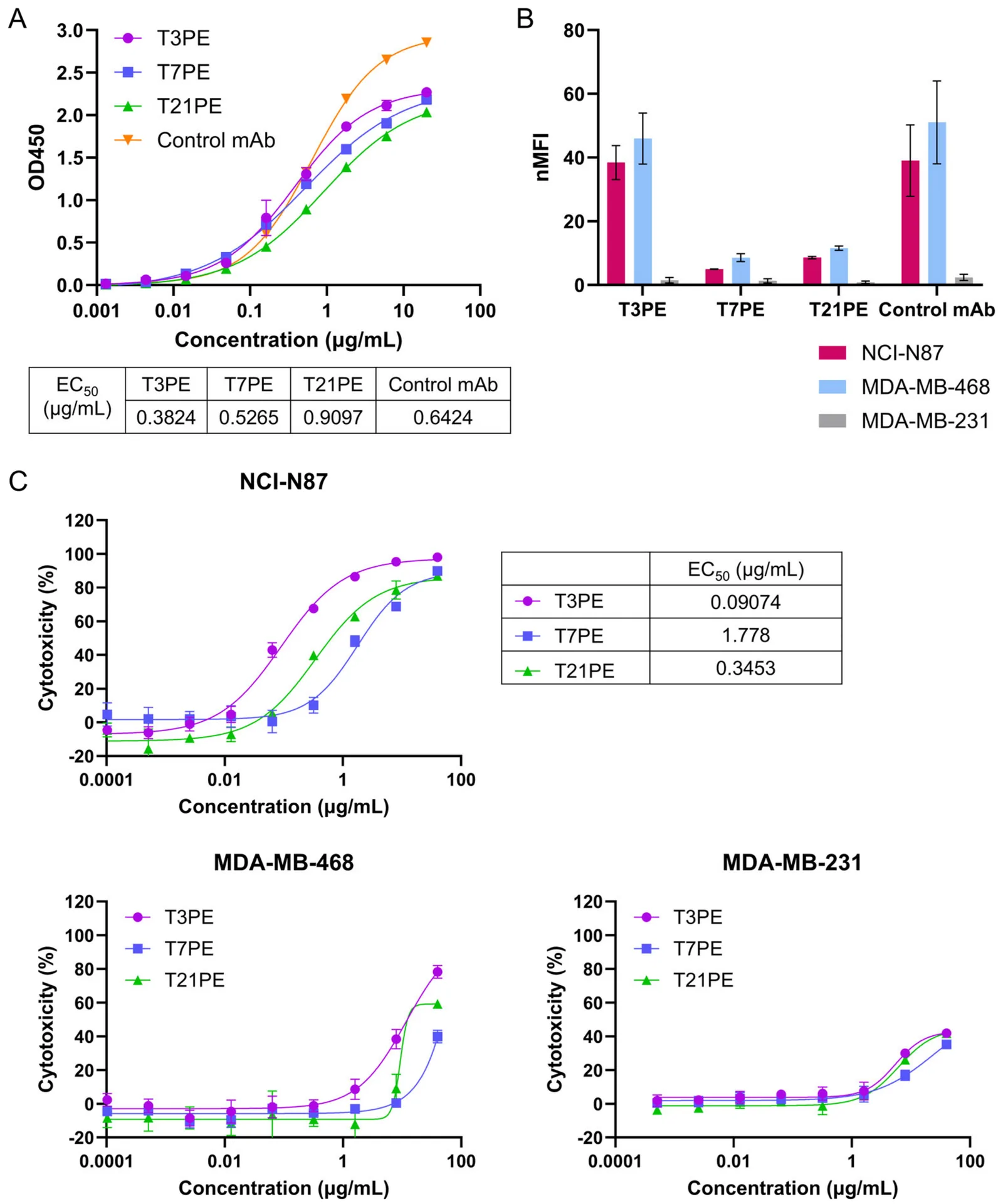
Binding properties and cytotoxic activities of the scFv-based anti-TROP2 immunotoxins. (**A**) ELISA binding curves of the immunotoxins and control mAb with corresponding EC_50_ values. (**B**) Relative TROP2 expression on three distinct tumor cell lines and cell binding activities of the immunotoxins evaluated by flow cytometry. Cells were incubated with 10 μg/mL mAb or immunotoxins and stained first with anti-PE antibody and then with AF488-conjugated anti-rabbit IgG (H+L) antibody. nMFI, normalized median fluorescence intensity. Data are presented as mean ± SEM. (**C**) In vitro cytotoxicity of the immunotoxins against distinct tumor cell lines detected by cell viability assay. Tumor cells were exposed to serial dilutions of immunotoxins for 72 h and cytotoxicity was determined by the WST-8-based Cell Counting Kit-8 (CCK-8) assay. Untreated tumor cells and cells treated with 100 μg/mL 5-fluorouracil (5-FU) served as controls for 100% and 0% viability, respectively. Sample readings were normalized to these controls.

To confirm that the immunotoxins retained target-binding capability after refolding, their binding activities were assessed using either recombinant TROP2 protein or cell lines with different TROP2 expression levels. The ELISA results showed that all three immunotoxins exhibited effective and dose-dependent binding to recombinant human TROP2 protein as the commercial rabbit anti-TROP2 mAb did, with EC_50_ values ranging from 0.3824 to 0.9097 μg/mL (Figure 2A). Next, three cell lines with varying TROP2 expression levels were selected and cell surface binding was evaluated by flow cytometry. MDA-MB-468 (TNBC cell line) and NCI-N87 (gastric cancer cell line) cells showed high surface expression of TROP2, whereas MDA-MB-231 (TNBC cell line) cells showed much lower TROP2 expression, evidenced by a minimal fluorescence shift when probed with the commercial rabbit anti-TROP2 mAb (Figure 2B). This is in line with previously reported quantitative measurements by flow cytometry: on average, 246,857 ± 64,651 TROP2 molecules per cell were detected on NCI-N87 cells, 301,603 ± 29,470 on MDA-MB-468 cells and 32,380 ± 5460 on MDA-MB-231 cells [31]. Notably, the immunotoxin T3PE exhibited strong binding to MDA-MB-468 and NCI-N87 which are TROP2-high-expressing cell lines, whereas substantially weaker binding was observed with the other two immunotoxins (T7PE and T21PE) (Figure 2B).

Subsequently, the cytotoxic activities of the immunotoxins were evaluated across the three aforementioned tumor cell lines (Figure 2C). T3PE induced dose-dependent cytotoxicity and exhibited superior killing potency to the others, particularly against NCI-N87 and MDA-MB-468 cells. The EC_50_ value of T3PE was approximately 20-fold and 4-fold lower than those of T7PE and T21PE in NCI-N87 cells. Based on the binding and cytotoxic activities, T3PE was selected for further studies.

### 2.3. Structure Modification and Stability Optimization of T3PE

**Figure 3:**
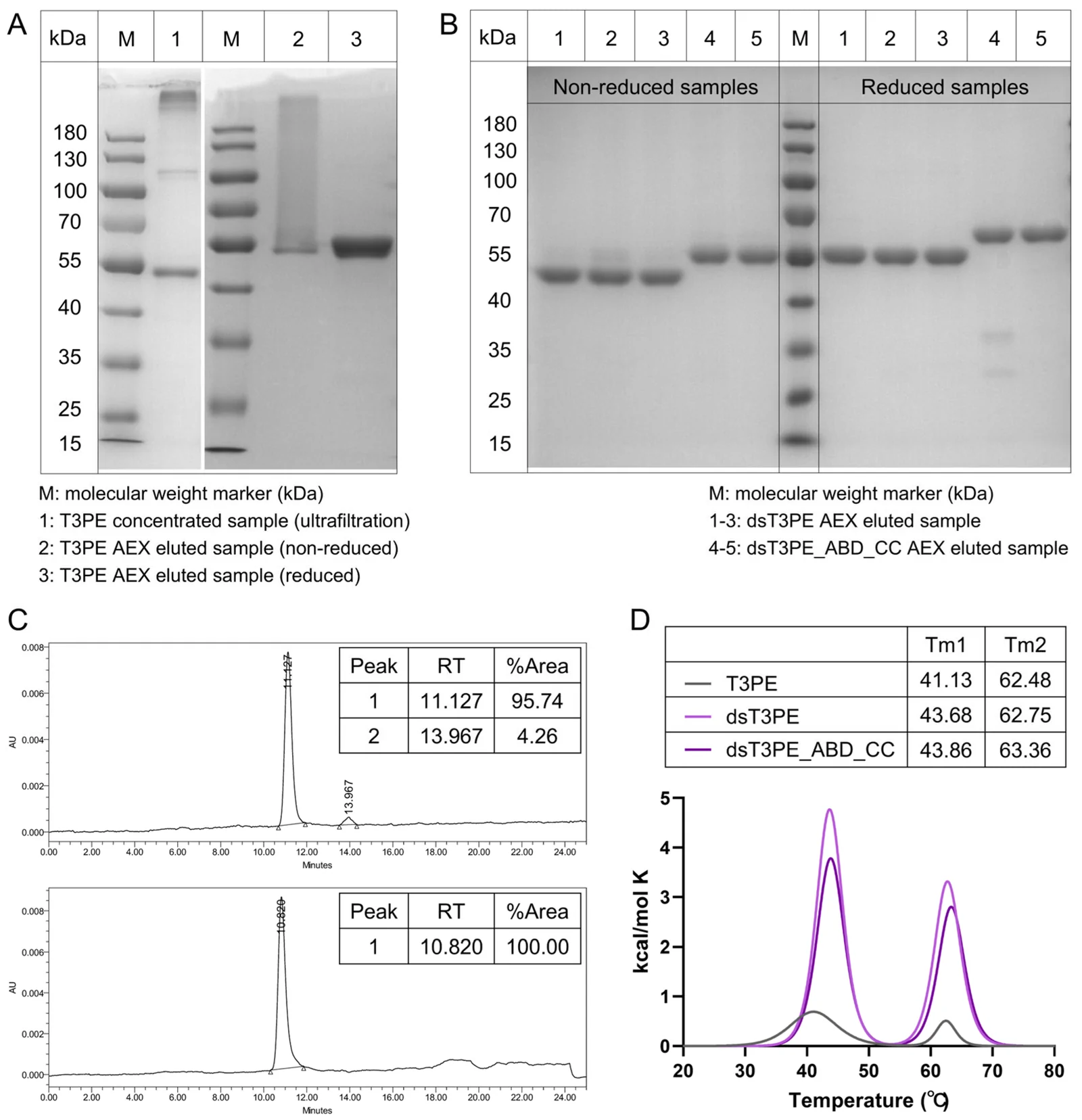
Disulfide bond stabilization of the immunotoxin-enhanced stability to physical stress during purification, prevented aggregation, and reduced degradation. (**A**) Aggregation was observed with T3PE after ultrafiltration and elution from chromatographic column analyzed by SDS-PAGE. M, molecular weight marker (kDa); 1, T3PE concentrated sample (ultrafiltration); 2 (non-reduced) and 3 (reduced), T3PE anion exchange chromatography (AEX)-eluted sample. (**B**) SDS-PAGE profiles and (**C**) SEC-HPLC chromatograms of purified dsT3PE and dsT3PE_ABD_CC after Q Sepharose chromatography. M, molecular weight marker (kDa); 1-3, dsT3PE AEX-eluted sample; 4-5, dsT3PE_ABD_CC AEX-eluted sample. The triangle symbols in (**C**) denote the start and end points used for peak integration. (**D**) Differential scanning calorimetry thermograms and Tm values of T3PE, dsT3PE and dsT3PE_ABD_CC.

Although the scFv format is preferred in immunotoxin design due to its compact size, suboptimal conformational stability often limits its therapeutic applications [32]. Consistently, our scFv-based T3PE exhibited a marked tendency to aggregate under physical stress, such as ultrafiltration and column elution (Figure 3A). To generate a stable scFv with favorable biophysical characteristics, an interdomain disulfide bond was introduced and the disulfide-stabilized T3PE was designated as dsT3PE (Figure 1A). Furthermore, another cysteine pair (CC) and an ABD were incorporated to improve the stability and prolong the circulation time of the immunotoxin, named dsT3PE_ABD_CC (Figure 1A). A single run through a Q Sepharose column was sufficient to obtain dsT3PE and dsT3PE_ABD_CC proteins with over 95% purity, as verified by SDS-PAGE and SEC-HPLC (Figure 3B,C). While a minor fraction of degradation products was detected for dsT3PE, as evidenced by peak 2 in the SEC-HPLC chromatogram, dsT3PE_ABD_CC appeared to have no detectable degradation, presumably due to the additional cysteine pair that mimicked the native disulfide bond in PE and prevented its degradation. Differential scanning calorimetry (DSC) analysis further confirmed that the two additional disulfide bonds in dsT3PE_ABD_CC conferred slight thermal stabilization, with a ΔTm1 of 2.73 °C for the scFv transition and 0.88 °C for the PE transition (Figure 3D).

**Figure 4:**
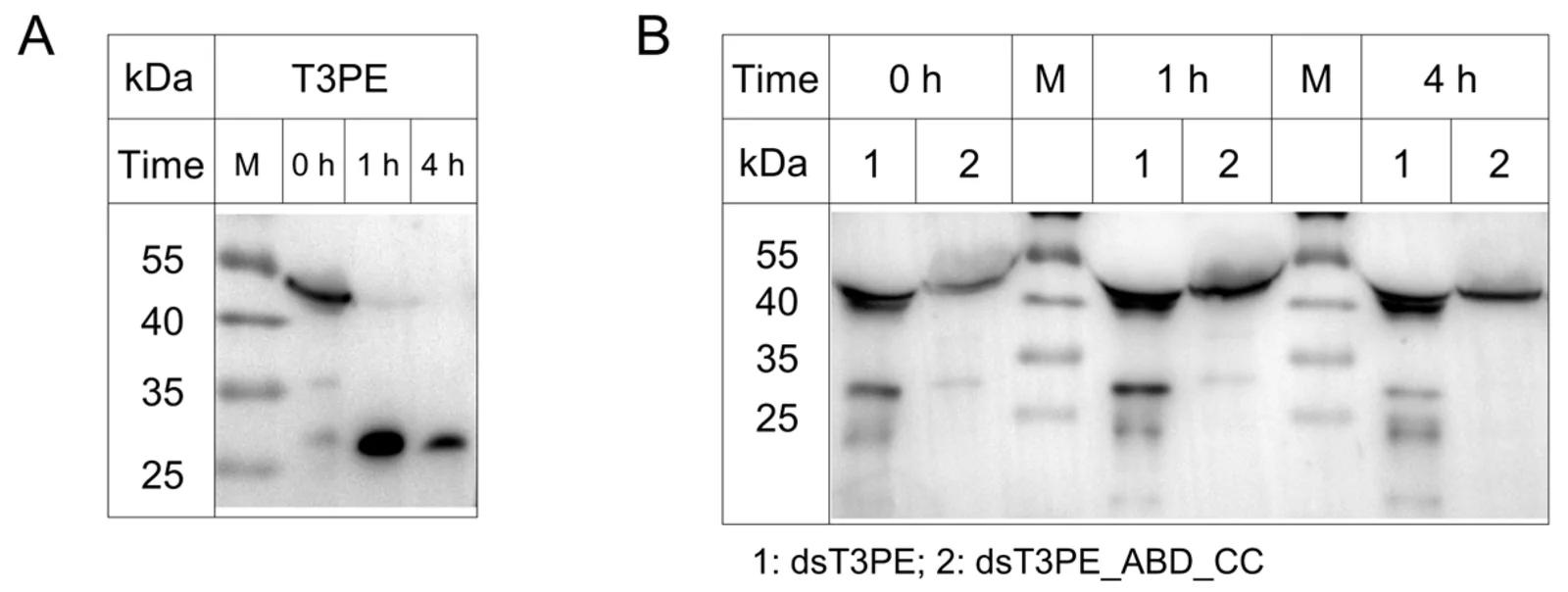
Disulfide bond stabilization of the immunotoxin in serum. Western blot analysis of (**A**) T3PE, (**B**) dsT3PE and dsT3PE_ABD_CC after being incubated in 100% mouse serum at 37 °C for 0, 1 and 4 h. 1, dsT3PE. 2, dsT3PE_ABD_CC.

Moreover, the engineered constructs also exhibited enhanced stability in 100% mouse serum (Figure 4 and Figure S2). While the original scFv-based immunotoxins, including T3PE, remained intact in PBS and cell culture medium with 10% FBS (Figure S2A), they were readily cleaved in 100% mouse serum, as evidenced by the disappearance of the intact ~50 kDa band and the production of a single band at ~25 kDa (Figure 4A and Figure S2B), indicating dissociation of the scFv and PE domains. This observation underscored the importance of evaluating immunotoxin stability under physiologically relevant conditions, as PBS and culture medium do not adequately replicate the proteolytic environment encountered in vivo. In contrast, both dsT3PE and dsT3PE_ABD_CC showed markedly improved serum stability, with the full-length immunotoxin remaining the predominant molecular form after 4 h incubation (Figure 4B). At time 0 h, dsT3PE had a relatively weak band at ~25 kDa which did not increase in intensity over time and could be the degradation product observed as the second peak in the SEC-HPLC chromatogram (Figure 3C), rather than a fragment formed during incubation.

### 2.4. Disulfide-Stabilized Immunotoxins Maintained Potent Cytotoxic Activities In Vitro

**Figure 5:**
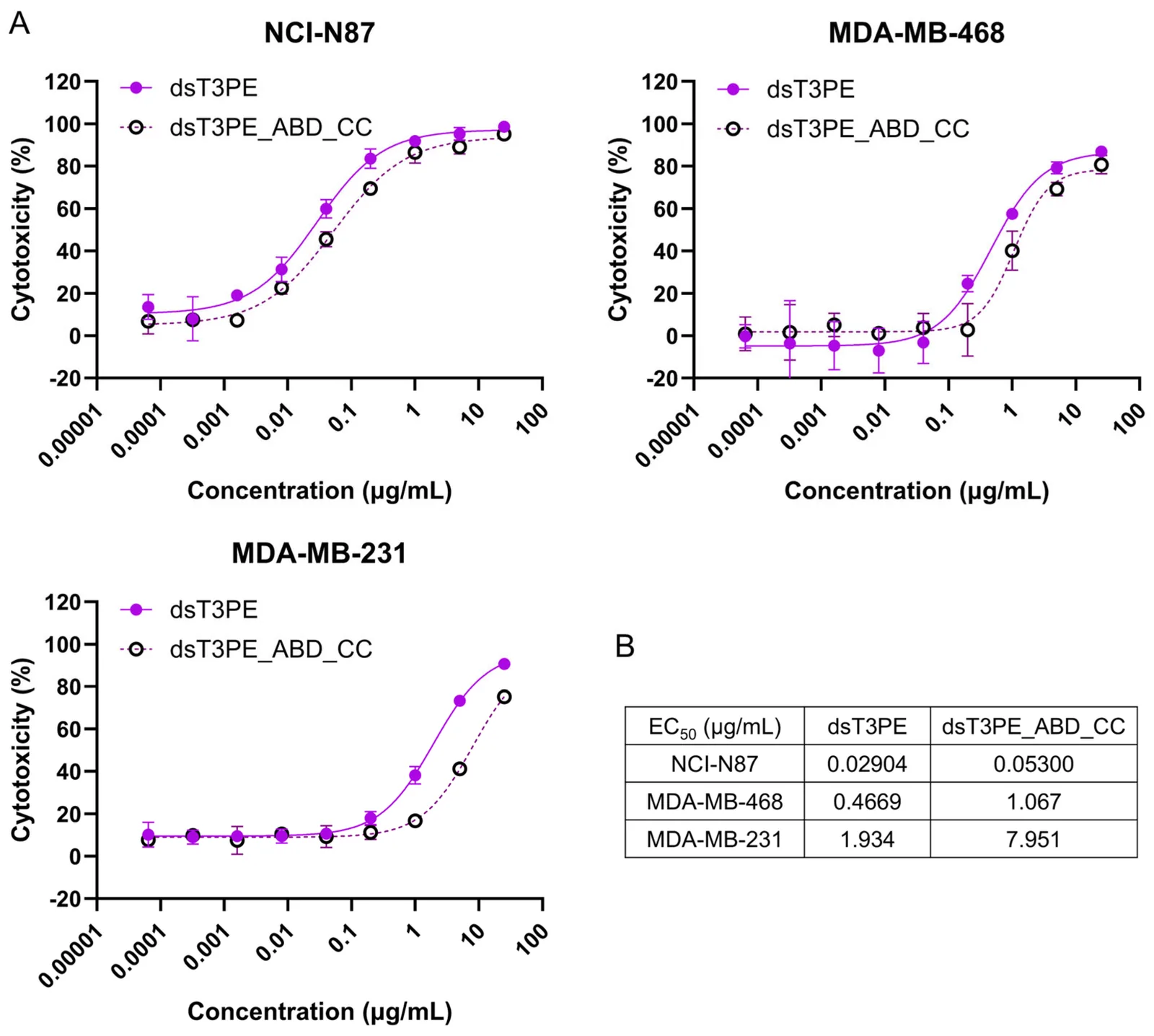
In vitro cytotoxic activities of dsT3PE and dsT3PE_ABD_CC. (**A**) Dose-dependent cytotoxicity of the immunotoxins dsT3PE and dsT3PE_ABD_CC against distinct tumor cell lines detected by cell viability assay. Tumor cells were exposed to serial dilutions of immunotoxins for 72 h and cytotoxicity was determined by the WST-8-based CCK-8 assay. Untreated tumor cells and cells treated with 100 μg/mL 5-FU served as controls for 100% and 0% viability, respectively. Sample readings were normalized to these controls. (**B**) EC_50_ values determined by cell viability assay.

The cytotoxic activities of dsT3PE and dsT3PE_ABD_CC were evaluated as described above. Both optimized immunotoxins exhibited potent and comparable cytotoxicity against NCI-N87 and MDA-MB-468 cells in the sub- to low nanomolar range (Figure 5). The EC_50_ values of dsT3PE and dsT3PE_ABD_CC against NCI-N87 were 0.5738 nM (0.02904 μg/mL) and 0.9161 nM (0.05300 μg/mL), which were approximately 3-fold and 2-fold lower than those of T3PE (Figure 2C). Notably, the enhanced potency was more evident in MDA-MB-468 cells, where the molar EC_50_ values decreased by more than 20- and 10-fold, respectively.

### 2.5. Cell Binding, Internalization, and Pharmacokinetic Profiles of the Immunotoxins dsT3PE and dsT3PE_ABD_CC

**Figure 6:**
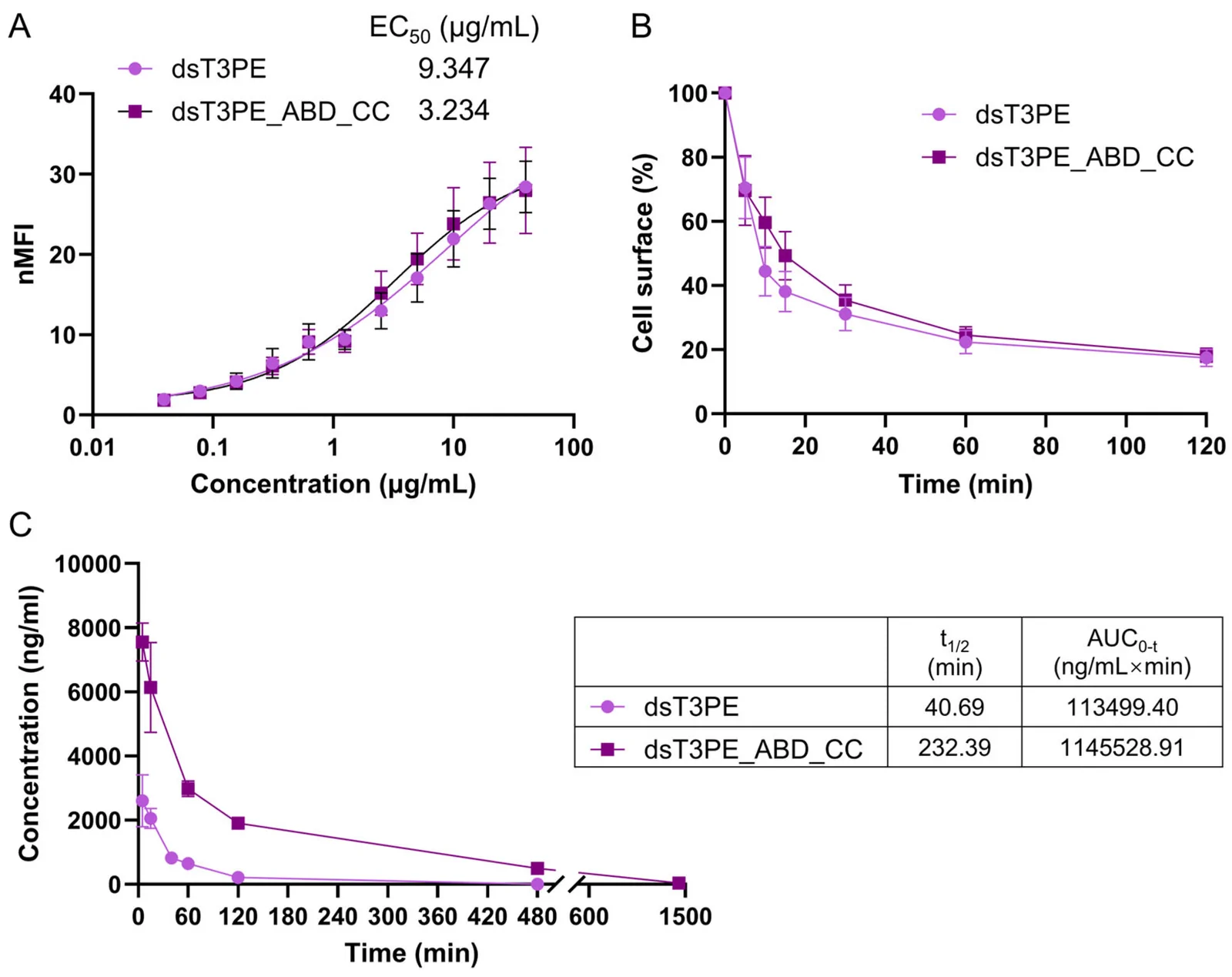
Binding, internalization, and pharmacokinetic (PK) characterization of dsT3PE and dsT3PE_ABD_CC in NCI-N87 cells and mice. (**A**) Dose-dependent binding of the immunotoxins to NCI-N87 cells measured by flow cytometry. Data were presented as mean ± SEM. (**B**) Internalization of the immunotoxins measured by flow cytometry. Time-dependent decrease in surface-bound immunotoxins on NCI-N87 cells at 37 °C. Data were presented as mean ± SEM. (**C**) Plasma concentration–time profiles of the immunotoxins following intravenous administration in mice (*n* = 4). Blood was sampled at the indicated time points and plasma immunotoxin concentrations were measured by ELISA. PK parameters, including terminal elimination half-life (t_1/2_) and area under the curve (AUC), were determined by non-compartmental analysis using PKSolver 2.0.

Prior to evaluating antitumor activity in vivo, the dose-dependent cell binding and internalization profiles of dsT3PE and dsT3PE_ABD_CC were characterized using NCI-N87 cells. Both immunotoxins exhibited effective and dose-dependent binding to TROP2-positive tumor cells, with EC_50_ values of 9.347 and 3.234 μg/mL, respectively (Figure 6A). Following cell binding, the immunotoxins were efficiently internalized into NCI-N87 cells, as determined by flow cytometry (Figure 6B). An average of ~50% of cell surface-bound immunotoxins were internalized within the first 15 min of incubation at 37 °C, and internalization reached a plateau by approximately 1 h.

Subsequently, the PK profiles of dsT3PE and dsT3PE_ABD_CC were evaluated to assess the contribution of the ABD to half-life extension (Figure 6C and Table S1). Following intravenous administration in mice, dsT3PE was rapidly eliminated from the systemic circulation, with a terminal half-life of 40.69 min. In contrast, incorporation of the ABD extended the plasma half-life of dsT3PE_ABD_CC by approximately 6-fold compared to dsT3PE. Consistently, the area under the curve (AUC) for dsT3PE_ABD_CC was 10-fold larger than that of dsT3PE, indicating substantially improved PK properties.

### 2.6. The Immunotoxins dsT3PE and dsT3PE_ABD_CC Exhibited Potent Antitumor Activities In Vivo

**Figure 7:**
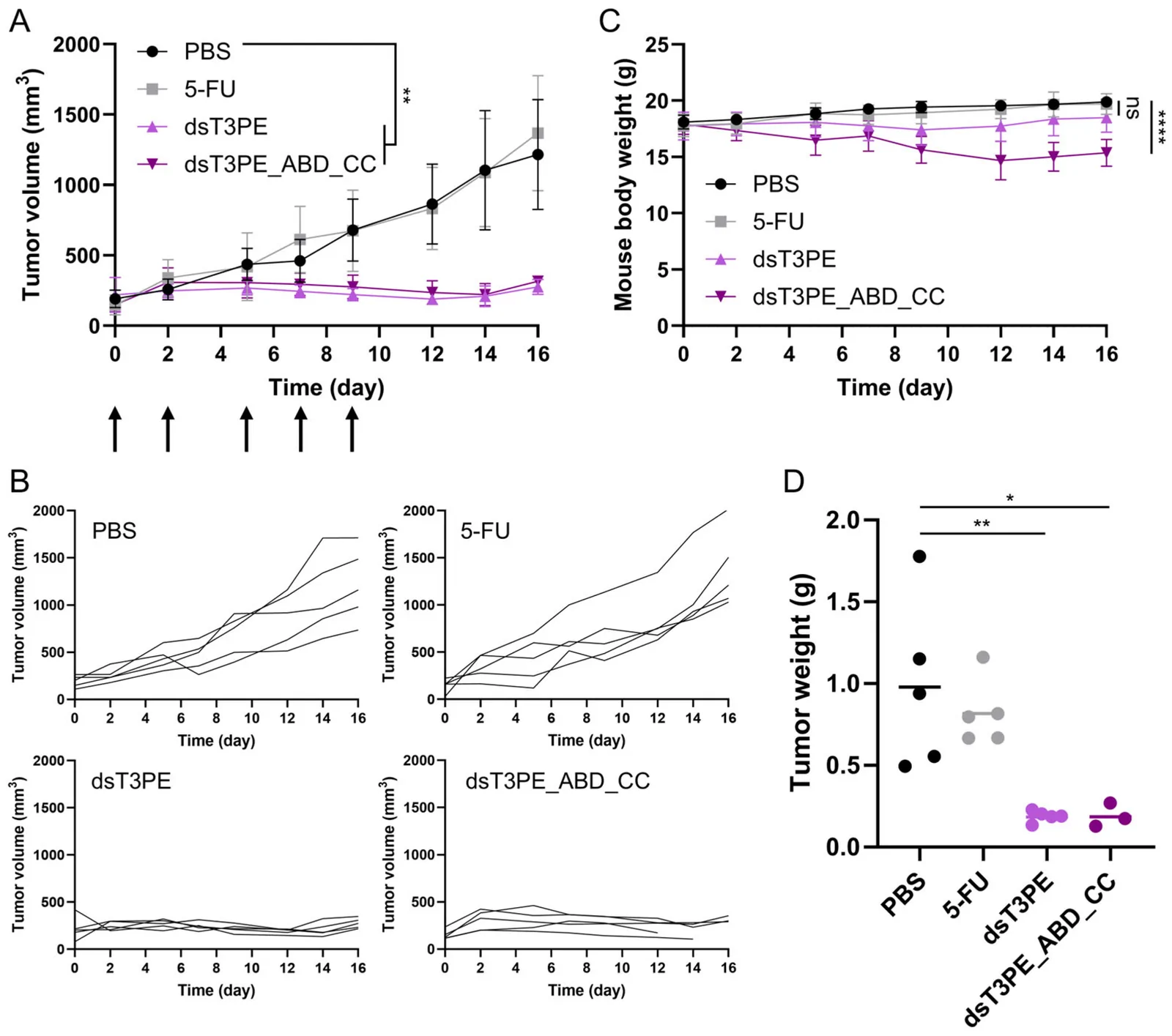
Antitumor activities of dsT3PE and dsT3PE_ABD_CC in an NCI-N87 xenograft mouse model. Five doses of PBS, 5-FU (10 mg/kg), dsT3PE (3 mg/kg) or dsT3PE_ABD_CC (3 mg/kg) were injected intravenously (*n* = 5). (**A**,**B**) Tumor volume and (**C**) mouse body weight were monitored three times per week. (**D**) Tumor weight was measured at the end of the study. Statistical significance was determined by unpaired one-way ANOVA with Tukey’s multiple comparison test (* *p* < 0.05, ** *p* < 0.01 and **** *p* < 0.0001; ns, not significant).

The antitumor activities of dsT3PE and dsT3PE_ABD_CC were next investigated using an NCI-N87 xenograft mouse model. 5-fluorouracil (5-FU), a widely used chemotherapeutic agent in gastric cancer, was included as a reference treatment. Both immunotoxins significantly suppressed tumor growth in this model, whereas 5-FU treatment showed no appreciable antitumor activity, with tumors progressing similarly to the PBS group (Figure 7). At the end of the study, the mean tumor weight and volume in both dsT3PE and dsT3PE_ABD_CC treatment groups were >70% lower than those in the PBS group (Figure 7A,D). The dosing regimen was well tolerated in the dsT3PE group; however, significant body weight loss was observed with the dsT3PE_ABD_CC treatment, indicating that the maximum tolerated dose was reached at this dosing schedule (Figure 7C).

## 3. Discussion

In this study, we designed and generated novel immunotoxins that were composed of fully human TROP2-binding antibody fragments and PE24 toxin. The antibody sequences were from a phage display library and predicted to bind three separate epitopes on recombinant human TROP2. Since epitope preference can influence antibody-mediated internalization and antitumor efficacy [33], we included representative clones from different epitope groups for evaluation. ScFv is one of the smallest functional elements of a native antibody and is widely used for the development of multi-specific antibodies and immunoconjugates [34]. To minimize the molecular size of the RITs and potentially facilitate their better tumor penetration, we selected scFv as the targeting moiety and constructed three scFv-based anti-TROP2 immunotoxins for preliminary evaluation. Among the three constructs, T3PE exhibited strong binding activity (EC_50_ in the low nanomolar range as measured by ELISA) and the most potent cytotoxicity with low nanomolar EC_50_ against NCI-N87. However, the scFv-based immunotoxin showed aggregation tendency and limited stability in serum, prompting further structure modification to improve the therapeutic potential.

The instability observed in the initial scFv-PE24 construct can be partially attributed to two structural liabilities. First, the variable heavy (VH) chain and light (VL) chain interface in scFv is maintained solely by non-covalent interactions, which are prone to dissociation at physiological temperature. VH-VL dissociation may result in aggregation driven by the exposure of hydrophobic surfaces [32]. Second, the truncation of domain II in PE24 removes the native disulfide bond protecting FCS, rendering the construct susceptible to premature proteolytic cleavage and toxin release in serum [35]. We therefore introduced two complementary disulfide engineering strategies to stabilize the construct. In dsT3PE, the scFv was converted to a disulfide-stabilized scFv (dsFv) to stabilize the VH-VL interface as previously described [36], which minimized the immunotoxin degradation in mouse serum. Building on dsT3PE, dsT3PE_ABD_CC incorporated an ABD as well as a disulfide bond flanking the FCS. SEC-HPLC analysis of the purified product showed a markedly increased monomer peak with reduced degradation fragments compared with dsT3PE, indicating improved product homogeneity. DSC analysis showed that the dsFv conversion improved the thermal stability of the targeting domain, although the Tm remains below the desired threshold for clinical development. A recently described “stapling” approach that bridges the scFv linker and the variable domains via two disulfide bonds has been shown to raise Tm by an average of 10 °C across multiple antibodies without compromising binding affinity and functionality [37]. This approach could potentially be explored as an alternative strategy to further improve the thermal stability of the construct.

Efficient cellular internalization is a critical determinant of immunotoxin potency, as the toxin must reach the cytosol to inactivate protein synthesis. Both dsT3PE and dsT3PE_ABD_CC exhibited dose-dependent cell binding and fast cellular uptake. After one hour of incubation at 37 °C, only 20–25% of bound immunotoxins remained on the cell surface. Consistent with this, the immunotoxins showed potent in vitro cytotoxicity and in vivo antitumor efficacy. However, 5-FU did not show significant activity in vivo, which was in line with previous reports using comparable dosing regimens in the same model [38]. Notably, sacituzumab govitecan, the first FDA-approved TROP2-targeted ADC, demonstrated significant antitumor activity in the NCI-N87 gastric cancer xenograft model at 0.35 mg/mouse administered twice weekly for 4 weeks [31]. The TROP2-targeting immunotoxins in this study achieved robust antitumor efficacy in the same model, supporting the clinical relevance and the therapeutic potential of dsT3PE and dsT3PE_ABD_CC. Considering 5-FU did not serve as an optimal benchmark, a limitation of this study is that a direct head-to-head comparison with a validated positive control, such as sacituzumab govitecan, was not included. Future studies should incorporate this control to further evaluate the efficacy of these immunotoxins.

Compared with dsT3PE, dsT3PE_ABD_CC exhibited an extended plasma half-life from 40.69 to 232.39 min, representing a 6-fold extension. This extension is primarily attributed to the non-covalent interaction between ABD and endogenous albumin, which protects the immunotoxin from renal filtration and enables neonatal Fc receptor-mediated recycling [39,40]. However, the treatment of dsT3PE_ABD_CC might be associated with dose-limiting toxicity, as indicated by the reduction in mouse body weight. In terms of PK profile of the molecule, the 10-fold increase in AUC reflected its elevated systemic exposure, which could simultaneously enhance antitumor efficacy and off-target toxicity, consistent with the observed adverse effects. The fact that both dsT3PE and dsT3PE_ABD_CC had comparable antitumor activity under the current dosing regimen suggested that a reduced dose or less frequent administration would be better for dsT3PE_ABD_CC to mitigate the increased toxicity while maintaining therapeutic efficacy. Dose optimization studies will be necessary to define the maximal tolerated dose and an appropriate dosing schedule. Another consideration is that bacterial-derived ABD may introduce novel immunogenic epitopes. ADA formation during multiple cycles of treatment could accelerate drug clearance and efficacy loss, as observed for some PE-based immunotoxins [17]. Encouragingly, deimmunized ABD variants have been developed [41], representing a route for future optimization.

TROP2 is widely recognized as a promising therapeutic target in solid tumors. While anti-TROP2 ADCs have achieved clinical success, large molecular size (~150 kDa) may limit their penetration into solid tumors and the chemical conjugation process yields heterogeneous products with variable drug-to-antibody ratios. Moreover, resistance to anti-TROP2 ADCs has emerged, particularly through payload-related mechanisms including topoisomerase I mutations, upregulation of drug efflux transporters and activation of DNA damage repair pathways [42,43,44]. These limitations underscore the demand for TROP2-targeting therapeutics with distinct cytotoxic mechanisms. PE-based immunotoxins could inhibit protein synthesis by the ADP-ribosylation of elongation factor 2, which provides a mechanism complementary to topoisomerase I inhibition and might bypass the resistance mentioned above.

However, no TROP2-targeting immunotoxin has yet entered clinical development. At the preclinical level, 5G8-PE38, an anti-TROP2 immunotoxin has been explored previously, which fused a shark nanobody (VNAR) to PE38, and demonstrated antitumor activity in a breast cancer xenograft model [21]. However, PE38 is susceptible to proteolytic degradation and is associated with high immunogenicity and non-specific toxicity [12,15,45]. The other preclinically reported TROP2-targeting immunotoxin, Fab-PE24, was assembled using SpyCatcher-SpyTag technology and showed efficacy in gastric and lung cancer xenograft models [22]. This modular approach required separate expression and multistep purification, increasing the complexity of generation. Furthermore, the SpyCatcher-SpyTag domain remained covalently in the final product, which introduced an extra non-functional bacterial sequence and might worsen the immunogenicity issues. The present study mitigated these drawbacks by adopting a truncated and B-cell epitope-silenced 24 kD PE fragment (PE24), with the immunotoxins expressed as a single chain followed by one-step column chromatography. The deimmunized PE24 has been clinically validated in LMB-100, which exhibited reduced immunogenicity and a 3-fold higher maximum tolerated dose than the PE38-based SS1P (140 vs. 45 μg/kg) [17,46]. However, after repeated treatment cycles, ADAs still developed, suggesting that further deimmunization, such as T cell epitope removal [47], remains worthwhile for future research. Together with a cytotoxic mechanism distinct from current ADC payloads, dsT3PE and dsT3PE_ABD_CC may offer a complementary therapeutic option for TROP2-positive solid tumors, particularly in the setting of ADC resistance.

Prior to clinical translation of dsT3PE and dsT3PE_ABD_CC, several aspects warrant further investigation. First, although robust antitumor efficacy was observed in the NCI-N87 xenograft model, future studies should evaluate their in vivo activity across tumors with varying TROP2 expression levels to clarify the relationship between TROP2 expression and therapeutic response. Second, the xenograft model was established in athymic nude mice, which lack a functional human immune system. The immunogenicity of the constructs should therefore be assessed using human immune cells in vitro or humanized mouse models. Together with the additional stability optimization, further deimmunization, and dosing optimization discussed above, these studies will support the continued development of TROP2-targeting immunotoxins for cancer therapy.

## 4. Materials and Methods

### 4.1. TROP2 Antibody Identification from Phage Display Libraries

Screening for novel TROP2 antibody sequences was performed on in-house (Jecho Laboratories, Inc., Frederick, MD, USA) scFv fully human phage display libraries (JechoMabs), which contain diverse (>10^10^) antibody sequences. The antigen, recombinant human TROP2 extracellular domain, was biotinylated and subjected to magnetic bead-based solution panning. After three rounds of panning, positive clones were sequenced and cloned into mammalian expression vectors as scFv-Fc fusion constructs, which were transiently expressed by HEK-293F cells and purified by protein A spin columns. The binding specificity and affinity of these antibody sequences were assessed by flow cytometry on TROP2-positive or -negative cell lines and by binding signals to recombinant human TROP2 in an ELISA assay. Epitope binning was performed by a cross-competition assay using Octet (ForteBio, Menlo Park, CA, USA).

### 4.2. Construction, Expression and Purification of the scFv-Based Immunotoxins

The antibody VH chain and VL chain, obtained in the phage display, were connected in VH-VL orientation through a (GGGGS)_3_ linker to form the scFv. The scFv was then attached to the modified PE through a linker containing FCS. A deimmunized 24 kDa variant of PE was used in this study that mainly consists of catalytic domain III with seven amino acid substitutions that silence B-cell epitopes (R505A, R427A, R490A, R467A, D463A, R456A and R538A) [12]. Additionally, the C-terminal REDLK sequence of PE was changed to KDEL for increased cytotoxic activity [16]. The immunotoxin double-stranded gene sequences were synthesized by GenScript (GenScript Biotech, Nanjing, China), cloned into the pET30a expression vector and transformed in *E. coli* BL21 (DE3) cells.

Immunotoxins were prepared from bacterial inclusion bodies following a previously established method with modifications [5]. Briefly, protein expression was induced at an OD_600_ of around 1.0 with the addition of 0.1 mM IPTG at 37 °C. After 6 h, bacteria were harvested, and inclusion bodies were released by high-pressure homogenization and extensively washed with wash buffer (20 mM phosphate buffer, 0.1 mM EDTA, 100 mM NaCl, 0.5 M guanidine-HCl, pH 7.0). The first three washes included 1% Triton X-100, followed by three washes without detergent. Inclusion bodies were solubilized in denaturation buffer (20 mM phosphate buffer, 6 M guanidine-HCl, 0.5 M arginine, 10 mM DTT, pH 7.0) with incubation at room temperature for 2 h and the soluble fraction was obtained by centrifugation. Controlled renaturation of solubilized protein was achieved by 100-fold dilution in refolding buffer (20 mM Tris-HCl, 0.5 M arginine, 0.1 mM EDTA, 50 mM NaCl, 5 mM GSH, 1 mM GSSH, pH 9.0) and gentle mixing at 4 °C overnight. After the procedure, the refolded and purified immunotoxin was dialyzed against PBS. Protein concentration was determined by a BCA assay kit (Beyotime, Shanghai, China).

### 4.3. Construction, Expression, Purification and Characterization of the Optimized Immunotoxins

To enhance the stability and increase the half-life of the immunotoxin, two structure-optimized variants were designed. An interchain disulfide bond was first introduced in the scFv by exchanging two structurally non-essential amino acids (VL 100 and VH 44) to cysteine [36]. The original scFv sequence in the vector was replaced with dsFv using restriction enzymes NdeI/NheI. The second modification included the addition of an ABD and a cysteine pair flanking FCS in the linker sequence. The re-designed linker was attached to the dsFv sequence and placed between NdeI/MfeI restriction sites. Fragments were digested and ligated by T4 ligase according to the manufacturer’s instructions (Takara Bio, Kusatsu, Shiga, Japan).

The optimized immunotoxins were expressed and refolded as described above. To obtain a high-purity product, an additional chromatographic step using an anion exchange column (HiScreen Capto Q ImpRes, Cytiva, Marlborough, MA, USA) was added to the purification procedure. The refolding buffer was replaced by dialysis against the equilibration buffer (20 mM Tris-HCl, 0.1 mM EDTA, pH 7.4). The dialyzed protein was then loaded onto the column and washed and eluted with a linear gradient of elution buffer (20 mM Tris-HCl, 1 M NaCl, pH 7.4) on an AKTA Avant system (Cytiva, Marlborough, MA, USA). Target protein fraction was collected and dialyzed against PBS.

The purity of the immunotoxins was assessed by SDS-PAGE and SEC-HPLC. For SDS-PAGE analysis, 4 μg of protein sample was resolved on a 10% Tris-glycine polyacrylamide gel and stained with Coomassie brilliant blue. SEC-HPLC was performed on an Agilent HPLC system connected to a TSKgel G3000SWXL (7.8 mm × 30 cm) column (Agilent, Santa Clara, CA, USA). The column was equilibrated with 100 mM phosphate buffer (pH 6.7) containing 100 mM Na_2_SO_4_, and 5 μL of the sample was injected at a flow rate of 0.9 mL/min for 25 min. Chromatograms were recorded at 280 nm to assess the purity of the final product. Furthermore, the conformational stability of the immunotoxins was measured by a DSC using NanoDSC (TA Instruments, New Castle, DE, USA) at a concentration of approximately 1 mg/mL and a scan rate of 1 °C/min over the range of 20–80 °C. The two-state scaled model with two peaks was used to calculate Tm from background-subtracted heat capacity curves in NanoAnalyze.

### 4.4. Cell Culture

Cell lines were acquired from the Cell Bank of the Chinese Academy of Sciences (Shanghai, China) and grown in a humidified chamber at 37 °C and 5% CO_2_ in complete cell culture media containing 10% FBS (Gibco, Waltham, MA, USA), 1% GlutaMax (Gibco) and 100 U/0.1 mg per mL penicillin/streptomycin (Gibco). RPMI 1640 (Gibco) was used for the gastric carcinoma cell line NCI-N87. DMEM (Gibco) was used for the TNBC cell lines MDA-MB-468 and MDA-MB-231.

### 4.5. Evaluation of Immunotoxin Binding Activity by ELISA and Flow Cytometry

The binding activity of the immunotoxins to recombinant human TROP2 protein was evaluated by ELISA. Briefly, 96-well plates were coated at 4 °C overnight with 0.5 μg/mL target protein (Sino Biological, Beijing, China; 10428-H08H). After blocking with 2% BSA in PBST, serial dilutions of the immunotoxins were added and incubated at 37 °C for 1 h. For detection, washed plates were first incubated with rabbit anti-PE antibody (1:10,000, Sigma, St. Louis, MO, USA; P2381) and then with HRP-conjugated anti-rabbit IgG antibody (1:2000, Cell Signaling Technology, Danvers, MA, USA; 7074) at room temperature for 1 h. Signals were developed by the addition of OptEIA TMB Substrate (BD Biosciences, San Jose, CA, USA). The reaction was stopped by ELISA stop solution (Solarbio, Beijing, China) and the absorbance was measured at 450 nm (Tecan, Männedorf, Switzerland).

For evaluation of cell binding activity, a single cell suspension of 2×10^5^ cells per sample was incubated with the immunotoxins or a commercial rabbit anti-TROP2 mAb (Sino Biological, 10428-R001) at 4 °C for 1 h. For detection, washed cells were first incubated with rabbit anti-PE antibody (1:5000) and then with AF488-conjugated anti-rabbit IgG (H+L) antibody (1:500, Jackson ImmunoResearch, West Grove, PA, USA; 711-545-152) at 4 °C for 1 h. Control cells were incubated in buffer only and stained in the same way with detection antibodies. The background fluorescence was used to calculate a normalized median fluorescence intensity. Samples were analyzed using a CytoFlex flow cytometer (Beckman Coulter, Brea, CA, USA).

### 4.6. Evaluation of Immunotoxin Cytotoxicity by Cell Viability Assay

Cells were seeded in 96-well plates at a density of 5000 cells/well and allowed to adhere overnight. The next day, serial dilutions of immunotoxins were added to the cells and incubated for 72 h. Inhibition of cell proliferation was detected by Cell Counting Kit-8 (Dojindo, Shanghai, China) with absorbance measured at 450 nm. Values were normalized to untreated control cells (representing maximal cell viability, defined as 100% viability) and to 5-FU-treated cells (100 μg/mL, representing maximal cytotoxicity, defined as 0% viability).

### 4.7. Evaluation of Immunotoxin Stability in Mouse Serum by Western Blotting

The immunotoxins (15 μg/mL) were incubated in 100% mouse serum (Yeasen, Shanghai, China), cell culture medium with 10% FBS or PBS at 37 °C for 0, 1 or 4 h. Samples were next diluted in PBS and run on 10% Tris-glycine polyacrylamide gels. Proteins were transferred to a PVDF membrane (Immobilon; Merck, Billerica, MA, USA) and blocked with 5% non-fat milk in TBST. For immunodetection, the membrane was soaked with rabbit anti-PE antibody (1:20,000) and probed with HRP-conjugated anti-rabbit IgG antibody (1:10,000) at room temperature for 1 h. Positive bands were visualized by Enhanced Chemiluminescent substrate (NCM Biotech, Suzhou, China) and captured using an imaging system (Tanon, Shanghai, China).

### 4.8. Detection of Immunotoxin Internalization Efficiency in Target Tumor Cells by Flow Cytometry

To analyze immunotoxin internalization, NCI-N87 cells (2 × 10^5^ per sample) were first placed on ice and incubated with 10 μg/mL of immunotoxins diluted in ice-cold PBS with 2% FBS for 0.5 h. Next, cells were washed to remove unbound immunotoxins and placed at 37 °C for 0, 5, 10, 15, 30, 60 or 120 min. After sampling, cells were kept on ice until staining and sequentially stained with rabbit anti-PE antibody (1:5000) and AF488-conjugated anti-rabbit IgG (H+L) antibody (1:500) at 4 °C for 1 h. Samples were analyzed using a CytoFlex flow cytometer (Beckman Coulter).

### 4.9. Measurement of Immunotoxin Plasma Half-Life In Vivo

All animal experiments were performed according to the guidelines and under the approval of the Institutional Animal Care and Use Committee of Shanghai Jiao Tong University.

For evaluation of plasma half-lives, 6-week-old Balb/c female mice were purchased from Charles River Laboratories (Beijing, China) and intravenously injected with the immunotoxins (dsT3PE or dsT3PE_ABD_CC) at 2 mg/kg. Two groups of mice (*n* = 4) were sampled at three separate time points for each immunotoxin (six time points in total: 5, 15, 40, 60, 120, 480 min for dsT3PE, and 5, 15, 60, 120, 480, 1440 min for dsT3PE_ABD_CC). Blood was collected into heparinized tubes by puncture of the retro-orbital venous plexus and centrifuged to obtain plasma. Immunotoxin concentrations were determined by ELISA as described above. Plasma was diluted in PBS (1:100 or 1:50), and immunotoxin dilution series were used to generate standard curves to extrapolate unknown plasma concentrations. The terminal elimination half-lives were calculated using PKSolver 2.0 based on a non-compartmental model.

### 4.10. Evaluation of Immunotoxin Antitumor Activity in Mouse Xenograft Model

The in vivo antitumor activity of the immunotoxins was evaluated in the NCI-N87 mouse xenograft model. NCI-N87 cells (5 × 10^6^ cells in 100 μL per mouse) were subcutaneously injected into the right flank of 5-week-old female athymic Balb/c nude mice. When the average tumor volume reached approximately 150 mm^3^, mice were randomized into four groups (*n* = 5). The treatment regimen consisted of five doses administered via tail vein injection on days 0, 2, 5, 7 and 9. The vehicle control group received PBS, while the experimental groups received the immunotoxins (dsT3PE or dsT3PE_ABD_CC) at 3.0 mg/kg. 5-FU (10 mg/kg) was included as a reference chemotherapy group based on previously reported dosing regimens in gastric cancer xenograft models [48,49]. Mouse body weight was monitored and tumor dimensions were measured by a digital caliper three times per week. Tumor volume was calculated using the following formula: 0.5 × length × width^2^. When tumor size in control groups reached 2000 mm^3^, the experiment was terminated, mice were euthanized by CO_2_ inhalation, and tumors were removed to measure the weights.

### 4.11. Statistical Analysis

Data were presented as mean ± SD, unless otherwise specified in the figure legends. Statistical analyses were performed by GraphPad Prism 10 (GraphPad Software, Boston, MA, USA). Unpaired one-way ANOVA with Tukey’s multiple comparison test was used to determine statistical significance (* *p* < 0.05) among experimental groups.

## 5. Conclusions

In conclusion, we designed and generated three novel TROP2-targeting immunotoxins by fusing phage display-derived fully human scFvs to PE24. Among them, T3PE showed strong binding and cytotoxic activity, but limited stability. Structure modification, including disulfide bond introduction and ABD fusion, reduced its aggregation, improved serum stability and extended plasma half-life, while preserving potent cytotoxicity. Additionally, the optimized immunotoxins, dsT3PE and dsT3PE_ABD_CC, demonstrated significant antitumor efficacy in the NCI-N87 mouse xenograft model. Together, these results supported the therapeutic potential of dsT3PE and dsT3PE_ABD_CC for the treatment of TROP2-positive cancers and provided a basis for their further development.

## Data Availability

The original contributions presented in this study are included in the article/Supplementary Material. Further inquiries can be directed to the corresponding author.

## Supplementary Materials

The following supporting information can be downloaded at https://www.mdpi.com/article/10.3390/ph19101586/s1: Figure S1. Epitope binning and binding affinity of selected anti-TROP2 scFv-Fc candidates; Figure S2. Stability evaluation of scFv-based immunotoxins under various conditions; Table S1. Additional PK parameters of dsT3PE and dsT3PE_ABD_CC in mice.

## Abbreviations

The following abbreviations are used in this manuscript:

5-FU	5-fluorouracil
ABD	albumin-binding domain
ADAs	anti-drug antibodies
ADCs	antibody–drug conjugates
AEX	anion exchange chromatography
AUC	area under the curve
CCK-8	Cell Counting Kit-8
DSC	differential scanning calorimetry
dsFv	disulfide-stabilized scFv
FCS	furin cleavage site
mAbs	monoclonal antibodies
nMFI	normalized median fluorescence intensity
PE	*Pseudomonas* Exotoxin A
PK	pharmacokinetic
RITs	recombinant immunotoxins
scFv	single-chain variable fragment
t_1/2_	terminal elimination half-life
TNBC	triple-negative breast cancer
TROP2	trophoblast cell surface antigen 2
VH	variable heavy
VL	variable light
