# Supplementary material for: Discovery and Optimization of the Novel Immunotoxins Targeting TROP2 for Cancer Therapy
Source: Pharmaceuticals (Basel). 2026 Oct 7;19(10):1586. doi: 10.3390/ph19101586 (PMC13708540; doi:10.3390/ph19101586)
Supplement: Supplementary file 1 [file pharmaceuticals-19-01586-s001.zip › pharmaceuticals-4582722-supplementary.pdf]

Supplementary Materials for

Discovery and Optimization of the Novel Immunotoxins Targeting TROP2 for Cancer Therapy

Junyan Li [1,†], Tanja Poredoš [1,†], Shuyi Xu [1], Lei Wang [1], Haiyang Yin [2], Yali Yue [1], Junjun Liu [1], Tong Wu [1], Yanlin Bian [1], Yueqing Xie [2,3] and Jianwei Zhu [1,*]

[1] Engineering Research Center of Cell & Therapeutic Antibody, Ministry of Education, School of Pharmacy, Shanghai Jiao Tong University, Shanghai, 200240, China
[2] Jecho Institute Co., Ltd., Shanghai, 200240, China
[3] Jecho Laboratories, Inc., Frederick, MD, 21704, USA
* Correspondence: jianweiz@sjtu.edu.cn
† These authors contributed equally to this work.

Supplementary Figure

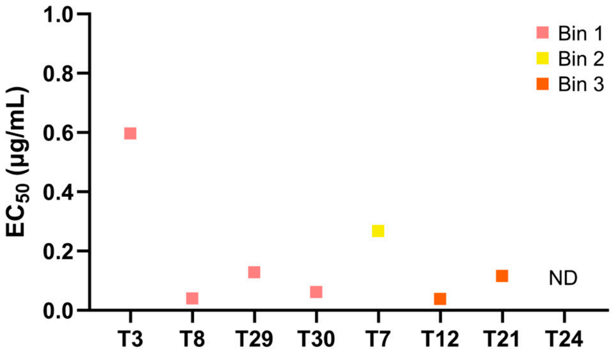

Figure S1. Epitope binning and binding affinity of selected anti-TROP2 scFv-Fc candidates. Each point represents the EC_{50} value of one candidate, which was determined by ELISA assay against human TROP2 protein. Colors indicate distinct epitope bins (pink, Bin 1; yellow, Bin 2; orange, Bin 3), which were identified by cross-competition assay using Octet. ND, not determined.

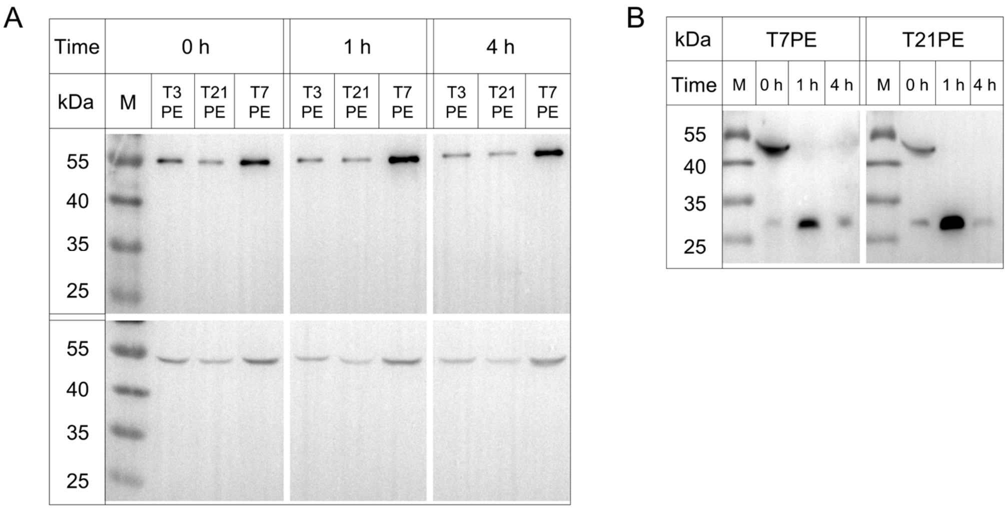

Figure S2. Stability evaluation of scFv-based immunotoxins under various conditions. Western blot analysis of the immunotoxins after incubated in (A) PBS (upper), cell culture medium with 10% FBS (lower), and (B) 100% mouse serum after incubated at 37°C for 0, 1 and 4 h.

Supplementary Table

Table S1. Additional PK parameters of dsT3PE and dsT3PE_ABD_CC in mice.

**Parameter**	**dsT3PE**	**dsT3PE_ABD_CC**
C_{max} (ng/mL)	2606.86	7557.31
$AUC_{0\text{-}inf}$ (ng/mL×min)	126153.93	1157105.21
$MRT_{0\text{-}inf}$ (min)	47.20	225.86
Cl (mL/min/kg)	15.85	1.73
V_{ss} (mL/kg)	748.29	390.39

The parameters were determined by non-compartmental analysis using PKSolver 2.0.
